# Behavioral Own-Body-Transformations in Children and Adolescents With Typical Development, Autism Spectrum Disorder, and Developmental Coordination Disorder

**DOI:** 10.3389/fpsyg.2018.00676

**Published:** 2018-05-25

**Authors:** Soizic Gauthier, Salvatore M. Anzalone, David Cohen, Mohamed Zaoui, Mohamed Chetouani, François Villa, Alain Berthoz, Jean Xavier

**Affiliations:** ^1^Département de Psychiatrie de l'Enfant et de l'Adolescent, AP-HP, Hôpital Pitié-Salpêtrière, Boulevard de l'Hôpital, Paris, France; ^2^CRPMS, EA 3522, Université Paris Diderot, Sorbonne Paris Cité, Paris, France; ^3^Equipe Berthoz, Collège de France, Paris, France; ^4^Laboratoire CHArt-THIM, EA4004, Université Paris 8, Saint Denis, France; ^5^Sorbonne Université, Institut des Systèmes Intelligents et de Robotique, CNRS UMR 7222, Paris, France

**Keywords:** behavioral own-body-transformations, developmental perspective, autism spectrum disorders (ASD), developmental coordination disorder (DCD), visuo-spatial abilities

## Abstract

**Background:** In motor imitation, taking a partner's perspective often involves a mental body transformation from an embodied, ego-centered viewpoint to a disembodied, hetero-centered viewpoint. Impairments of both own-body-transformation (OBT) and abnormalities in visual-spatial processing have been reported in patients with neurodevelopmental disorders including autism spectrum disorder (ASD). In the context of a visual-motor interactive task, studying OBT impairments while disentangling the contribution of visual-spatial impairments associated with motor coordination problems has not been investigated.

**Methods:** 85 children and adolescents (39 controls with typical development, TD; 29 patients with ASD; 17 patients with developmental coordination disorder, DCD), aged 6–19 years, participated in a behavioral paradigm in which participants interacted with a virtual tightrope walker (TW) standing and moving with him. The protocol enables to distinguish ego-centered and hetero-centered perspectives.

**Results:** We show that (1) OBT was possible but difficult for children with neurodevelopmental disorders, as well as for TD children, when the task required the participant to perform a mental rotation in order to adopt a hetero-centered perspective. (2) Using multivariate models, hetero-centered perspective score was significantly associated with age, TW orientation, latency, and diagnosis. ASD and TD groups' performances were close and significantly correlated with age. However, it was not the case for DCD, since this group was specifically handicapped by visual-spatial impairments. (3) ASD and DCD did not perform similarly: motor performance as shown by movement amplitude was better in DCD than ASD. ASD motor response was more ambiguous and hardly readable.

**Conclusion**: Changing perspective in a spatial environment is possible for patients with ASD although delayed compared with TD children. In patients with DCD, their visual-spatial impairments negatively modulated their performances in the experiment.

## Introduction

Imitation plays an essential role in the development of intersubjectivity (Nadel and Potier, 2002) and is involved in self-building (Meltzoff, 2005). Studies of imitation provide evidence that from birth, observation and execution of human actions are innately coupled (Meltzoff and Decety, 2003). Spontaneous imitation among children reveals a playful dynamic through a succession of symbolic and spatial viewpoint changes (Xavier et al., 2013). This form of interaction, involving visual perspective transformations, requires one to map one's own behavior onto the behavior of another. Taking the other's perspective relies on an own-body-transformation (OBT), i.e., a mental spatial transformation of the first-person perspective, corresponding to an egocentric viewpoint (Vogeley and Fink, 2003) with the possibility of spontaneous change in self-location, from embodied (ego-centered) to disembodied (hetero-centered) perspective, in order to adopt a third-person perspective (Decety and Grèzes, 2006). The primacy of the self-perspective is more tightly coupled to the sensory-motor system than the third-person perspective which requires additional visual-spatial transformation (Jackson et al., 2006). Lambrey et al. (2011) demonstrated the existence of separate brain networks involved in egocentric and allocentric perspectives.

According to Piaget and Inhelder (1997), children are able to move from an egocentric point of view from the age of 7 (Lange-Küttner, 2009) to represent and coordinate multiple perspectives in one coherent spatial framework. Perspective-taking (PT) requires distinguishing: (i) the visual PT level 1 (VPT1), acquired between the ages of 18 and 24 months (Moll et al., 2007), which is the ability to judge what a person can and cannot see, and (ii) visual PT level 2 (VPT2), acquired around 4–5 years, which is the ability to understand that two different people viewing a scene or object simultaneously do not necessarily see objects in the same way (Flavell et al., 1981). VPT2 requires an embodied spatial transformation of the participant's viewpoint to that of the target (Surtees et al., 2013). Abnormalities in OBT have been described in neurodevelopmental disorders such as developmental coordination disorder (DCD) and autism spectrum disorder (ASD). Children with DCD display motor incoordination and visual-spatial processing deficits (Mazeau, 2000), which may affect their imitation abilities (Werner et al., 2012). These visual-spatial impairments underlie the difficulties that these children face to perform imagined transformations from an egocentric perspective. These difficulties appeared more pronounced for severe DCD (Williams et al., 2008). Children with ASD constitute a heterogenous group in which all individuals present impaired social interaction as core symptom. They can display motor coordination impairments (Fournier et al., 2010) and atypical visual-spatial processing (Caron et al., 2006; Damarla et al., 2010). This atypicality is expressed through a dichotomy between enhanced visual-spatial abilities in tasks necessitating local and static information processing (Mitchell and Ropar, 2004; Mottron et al., 2006; Muth et al., 2014) and impaired performances regarding global and dynamic information analysis (Bertone et al., 2005). Alongside impairments in understanding other's mental states (Frith, 2012), individuals with ASD also have difficulty with VPT. Most studies exploring VPT suggest that whilst VPT1 is intact in people with ASD, VPT2 may be impaired (Pearson et al., 2013), due to the fact that the ability to perform OBT (i.e., egocentric transformations), contrary to the ability to perform mental rotations (object-based transformations), is deficient in people with ASD (Pearson et al., 2014). In either typical developing (TD) children or children with ASD, most of the studies did not investigate OBT behaviorally, that is, to say, involving body movements while an individual is interacting with another. Instead, they used laterality judgment tasks, participants in a seated position, looking at a visual display. This may have important implications for the task's interpretation. Using the Magic Carpet, a computerized navigational version of the traditional (paper/manual) Corsi Block-tapping Test, in order to explore spatial memory, Belmonti et al. (2014) showed a dissociation between a reaching mode (near) and a navigational (extra-personal) mode, each involving specific cognitive strategies and brain networks.

The developmental aspects of OBT in children with ASD and DCD have not yet been addressed in a semi-ecological task such as (Thirioux et al., 2009) experimental setup. This motor imitation task, based on an interaction with a tightrope walker avatar, investigates whether participants can embody another person's behavior and, if so, whether they do it with either an embodied or disembodied self-location (see section Method).

In order to explore OBT from a developmental viewpoint, we examined imitation abilities by means of this interaction paradigm. To better understand the OBT difficulties in children with ASD, we compared the potential alteration of the development of OBT abilities with those of the TD control group in the context of a visual-motor interactive task. Furthermore, to disentangle the contribution of visual-spatial impairments associated with motor coordination problems in ASD children, we explored the potential alteration of the development of OBT in a group of children with DCD. The following hypotheses were made: (1) within the TD group, there will be an age effect on OBT abilities and quality of movements. This age effect will be explored in the ASD and DCD groups. (2) When compared to TD children, children with ASD will show significant impairments in terms of both OBT abilities and quality of movements. (3) When compared to TD children, children with DCD will also show significant impairments in terms of both OBT abilities and quality of movements, but to a lesser extent compared to ASD.

## Methods

### Participants

A total of 79 children and adolescents, aged 6–19 years, were recruited in the Department of Child and Adolescent Psychiatry of the Pitié-Salpêtrière University hospital. We included a control group with typical development (*n* = 36) to allow the study of spatial rotation age threshold, a group of children with ASD (*n* = 25), and a group of children with DCD (*n* = 14). For each participant, we checked their ability to understand the imitative task (see section Paradigm). Exclusion criteria were ongoing medical conditions (e.g., seizures, sensory deficit) and severe language impairment that can be comorbid with ASD and DCD. For each patient, the diagnoses were based on all available information (including direct interviews, family history data, and records) and according to the Diagnostic and Statistical Manual of Mental Disorders-Fifth Edition criteria (American Psychiatric Association, 2013). Each ASD patient was also given a series of clinical assessments: the Autism Diagnostic Interview-Revised (ADI-R) (Rutter et al., 2013) was used to score autism core symptoms; the cognitive quotient was ascertained by using the WISC-IV (Wechsler Intelligent Scale for Children-IV) (Wechsler, 2003). Children with DCD were evaluated during a psychomotor assessment that included quantitative testing (e.g., the Movement Assessment Battery for Children, M-ABC) (Henderson et al., 2016) performed by an occupational therapist. WISC-IV was also performed in DCD patients. Among the 26 ASD participants up, 15 had clinical motor impairments and also performed M-ABC. Developmental age was calculated on the WISC-IV basis. Each patient (ASD or DCD) was matched, according to developmental age, with a healthy TD child using chronological age, assuming that for TD children, chronological and developmental age were equal. Regarding gender, we decided to recruit 1 TD girl for 2 TD boys to follow the unequal sex ratio known in neurodevelopmental disorders. We decided to avoid strict matching to have a sufficient number of girls in the TD group to allow exploring a possible gender effect in the multivariate models (see section Statistical). The TD children recruited were children of staff members of the child and adolescent department of the Pitié-Salpêtrière hospital and of the laboratory *Institut des Systèmes Intelligents et de Robotiques*, who attended typical schooling for which the chronological age corresponds to the developmental age. Including written informed parental consent, the study was specifically reviewed and approved by an ethics committee, the CERES (Comité d'Ethique de la Recherche en Santé) [N° IRB: 20150700001072]. Demographics and clinical characteristics of the 79 participants with exploitable data are given in Table 1.

**Table 1:** Main characteristics of the participants.

	**ASD (*N* = 26)**	**DCD (*N* = 15)**	**TD (*N* = 38)**
Chronological age, mean (±*SD*) Male/Female	12.65 (3.66) 21/5	12.17 (3.38) 9/6	12.03 (4.11) 23/15
**WISC-4**, mean (± *SD*) Verbal comprehensive index Perceptual organization index Working memory index Processing speed index Developmental age (IQ ^*^ age /100)	94.33 (30.60) 88.30 (28.69) 80.57 (25.73) 73 (17.23) 11.75 (5.08)	98,69 (17.54) 92.92 (17.70) 85.72 (17.89) 84.54 (17.81) 11.61 (3.23)	Non relevant 12.03 (4.11)
**ADI-R scores**, [mean (±*SD*), min, max] Social impairment Verbal communication Restricted, repetitive behaviors	14.87 (6.86), 5, 30 10.04 (5.35), 5, 23 3.83 (3), 1, 12	Non relevant	Non relevant
**VABS for Children**, mean (±*SD*) Communication domain Social autonomy Socialization domain Interpersonal relationships Play and leisure time Coping skills	28.52 (14) 30.73 (11.46) 24.31 (12.03) 17.36 (7.49)	Non relevant	Non relevant
**M-ABC**, mean (±*SD*)	−3.02 (2.50)^*^	−1.49 (1.60)	Non relevant

*ASD, autism spectrum disorder; DCD, developmental coordination disorder; TD, children with typical development; ADI-R, autism diagnostic interview – revised version; VABS, Vineland Adaptive Behavior Scales; WISC-4, Wechsler Intelligence Scale for Children (version 4), M-ABC, movement assessment battery for children*.

* *M-ABC scores in children with ASD are only available for the children with clinical motor impairments (N = 15)*.

### Paradigm

#### The tightrope walker paradigm

**Figure 1:**
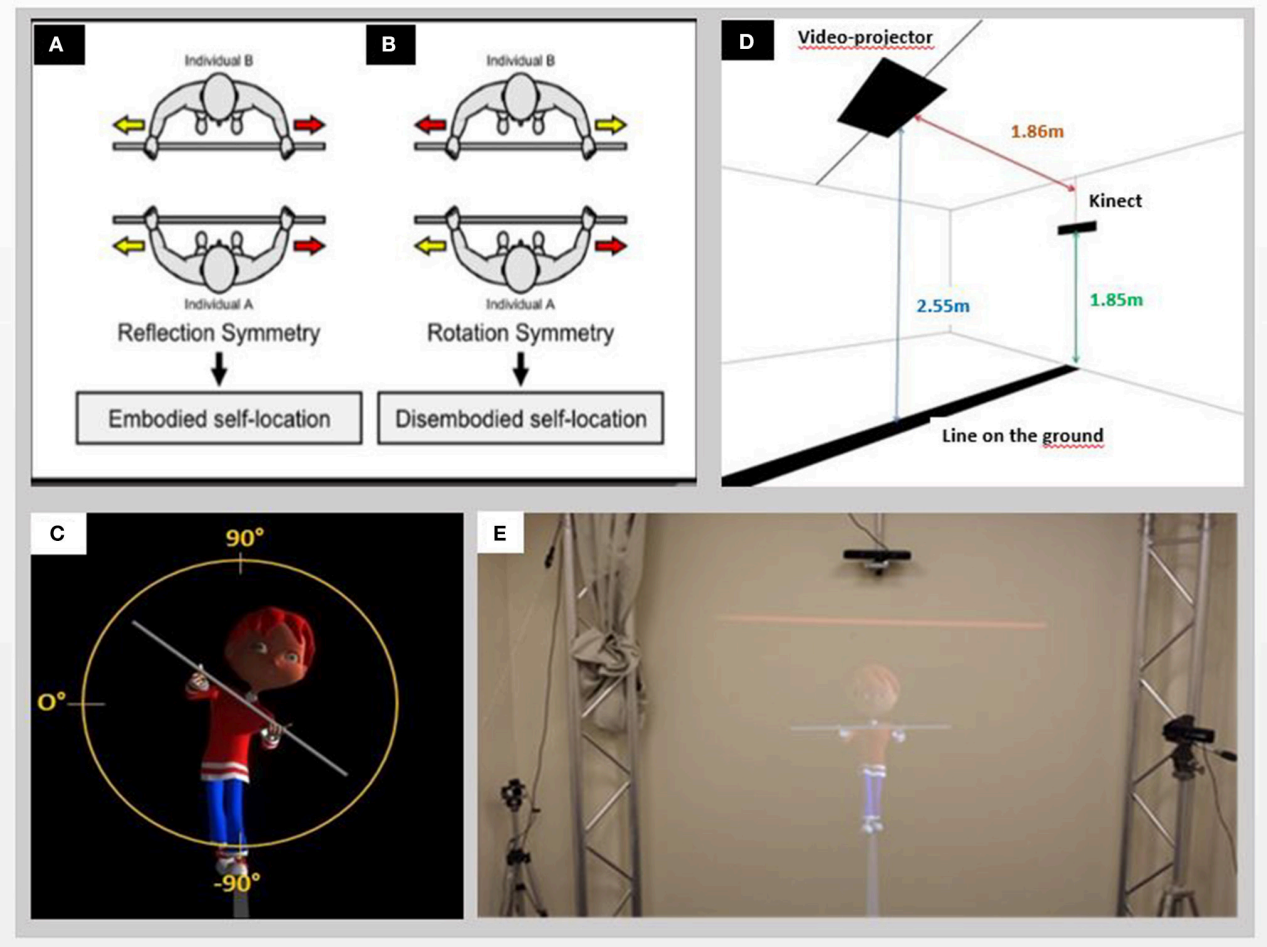
Principles and set-up of the experiment: **(A)** Reflection and **(B)** rotation symmetry during face-to-face interaction; **(C)** Tightrope walker's bar inclination measurement; **(D)** Schematic illustration of the experimental room; **(E)** Projection on the wall of a tightrope walker avatar. The roll angle of the participants' head and of the participants' bar in the frontal plane were recorded. The roll angles of the head and bar of the avatar were also recorded. **(A,B)** Courtesy of (Thirioux et al., 2010).

The tightrope walker paradigm is an experimental setup designed in order to test the ability to change of spatial viewpoints in a spontaneous motor task (Thirioux et al., 2009, 2010). During self–other interaction, it enables one to test the ability to (1) inhibit one's ego-centered perspective and (2) adopt the visual- spatial perspective of others (see Figures 1A,B). When two individuals, A and B, are facing each other, if B is leaning to his right, then A can imitate B's movement by leaning either to his left or to his right. If A is leaning to his left when B is leaning to his right, then A is mirroring B's movement. This “reflection symmetry” indicates that A is not picturing B's point of view, keeping his own ego-centered perspective. This is called embodied self-body location. But, if A is leaning to his right when B is also leaning to his right, then A reproduces B's movement by using “rotation symmetry.” This indicates that A is imagining himself at the B's body position. This is called disembodied self-location: when the imagined and transformed extra-personal position of one's own-body does not match the spatial position of one's physical body (Blanke, 2012). Then, A adopts a hetero-centered perspective.

#### The “funambule” tightrope walker paradigm in our experimental setup

We adapted the paradigm of the “funambule” tightrope walker to children by adjusting the size and giving it a cartoon aspect of a child. We developed a 3D animation where a 3D character is walking on a rope and holding a bar in front of him (Figure 1C). The 3D animation was developed under Unity—a 3D engine used for virtual reality. The application runs on a PC under Windows 7. The natural movements of the 3D character come from a series of motion capture with the 12 cameras of VICON system. The animated tightrope walker (TW) walking on a rope was displayed life-sized by a rear-projector onto a large screen (2 m^*^2 m). It was 0.81 meter-high when standing in the middle part of his rope, 1.13 meter-high when he was the “closest” to the participant. To mimic everyday social encounters and to reinforce interactions giving participants the impression to act in the same spatial environment as the TW, participants stood on a black line (2 m/10 cm; length/width) which prolonged on the ground as the avatar's rope on the screen (Figure 1D). Before the movie started, we asked participants to find a comfortable position, legs slightly apart and not to shift from their position in response to the moves of the TW. For each participant, we checked the ability to understand the motor imitation task: the participant was asked to imitate the clinician who stood in front of him holding a wooden bar and tilting it on both sides. Participants held a wooden bar (length: 1 m) horizontally in front of them. In order to reinforce both the interaction with the TW and the ecological features of the task, the TW's forward and backward movements' durations were randomized.

### Protocol and tasks

First, the tightrope walker (TW) was shown in a front-facing orientation, standing with his right foot in front of the left on the rope for the first 30 s. Then, during 7 following trials, numbered from 1 to 7, the TW, walking successively either forward either backward, was alternatively shown in two orientations: (i) a front-facing orientation when the TW walks forward; imitating him in this condition required a perspective change. (ii) A back-facing orientation when, the TW walking backward, the participants saw it from his back. While walking, for each orientation, the TW executed lateral tilts with his bar in random order either to his right or to his left (Figure 1E), with a maximum amplitude of 51° (mean amplitude: 44°) and a maximum duration of 3.2 s (mean duration: 2.7 s). Each trial lasted 35.7 s and was composed of 7 TW's tilts. For half of the participants in each group, the first trial presents the TW front-facing, and for the other half, the first trial presents the TW back-facing. The test was divided in two parts: first, a semi-no instruction task (trial 1 and 2) and second, an own-body- transformation guided task (trial 3–7).

#### No instruction task (NI task)

During trials 1 and 2, the TW makes 2 walks, one forward (front-facing oriented) and one backward (back-facing oriented) from the participant. Participants were instructed to observe the tilts of the TW and to lean when he was leaning. We consider this condition as spontaneous insofar no instruction with respect to direction of leaning was given.

#### Own-body-transformation guided task (OBT guided task)

During trial 3–7, the TW made 5 walks, either 2 forward and 3 backward or 3 forward and 2 backward walks. Participants were also instructed to observe the tilts of the TW and to lean when he was leaning, but additionally explicitly to imagine their body in the position of the TW's body: “you're going to imagine you are the TW; you have to put yourself in its shoes.” This additional instruction aimed driving participants to adopt a hetero-centered perspective.

### Data recording and metrics

#### Data recording

Participants' movements in the frontal plane were recorded by a KINECT device, located in front of them, on the wall above the TW, at a height of 1.85 m. The KINECT captured the figure of the participant at a mean rate of about 25 frames per second. The information contained by each frame were accessible through a comma-separated values (CSVs) file. For each frame, the participant posture and the TW posture were recorded as well as the timing of the frame, the participant's and the TW's bars and head inclinations were measured in degrees (Figure 1C).

#### Characteristics of movements

Each participants' and TW's movements were extracted and recorded for offline analysis and labeling (see Supplementary Material “Examples of recorded participants movements and tightrope walker movements”, Figures S1–S8). Automatic approaches for events recognition and data annotation have a high margin of error when they are applied to noisy information. This is particularly true while dealing with behavioral data from people with physical or cognitive disabilities. We, therefore, opted for a systematic, manual annotation of children's movement trajectories employing a software we developed that enabled us seeing the curves, selecting specific frames on it and labeling events in the experiment timeline (see Supplementary Material, Figures S4–S6).

In previous studies (Thirioux et al., 2009), responses were determined by a bar angle getting higher or lower than 5 or −5°. However, we could not do exactly the same in our study because the trajectory curves of some participants indicated that they did not always come back to their initial position (around 0°) after a movement. Moreover, some participants swung regularly their bar with a swing amplitude higher than 5° between two movements, whereas some other stood very still (see Figure S2). Consequently, our definitions could not rely on this 5° rule and we had to define the proposed events without a strict degree limitation. In some cases, it was not possible to annotate all the movements due to the inability of the participant to imitate the tightrope (see Figure S7).

We characterized three types of annotated movements: (a) a *mirroring response* according to an ego-centered perspective; (b) a response adopting the perspective of the TW (according to a *hetero-centered perspective*), and (c) we qualified some responses as mixed to the extent that the two responses (a) and (b) were combined, the participant first using one of them before correcting himself and performing the other (see Figure S8 in Supplementary Material).

For each curve, we distinguished those we were able to annotate (“tagged”) from those we could not (“not tagged”). Each “tagged” event is a movement of the participant and was defined by its beginning, its peak, and its end (see Supplementary Material Table S1). Considering the corpus of “tagged” participants' movements (TPM), we determined their types (i.e., ego-centered, hetero-centered, or mixed). Two different raters performed the manual annotation of the curves and Cohen's Kappa coefficient was used to test their agreement. We found an excellent inter-rater reliability for both the number of tagged movements and the type of strategies (ego-centered or hetero-centered) with, respectively, *k* = 0.91 and *k* = 0.94. We also determined a set of quantitative measures characterizing them: the amplitude, the length, the latency, the overshoot, and the overlength. The measures defined have been employed to compare children's movements with those of the TW. All variables' definitions are given in Table 2.

**Table 2:** Definitions of the types of response on the bar trajectory curve and variables used in the analysis.

**Response type name**	**Response type definition**
Not tagged movement	Movements that are not labeled, in the following cases: • One of the parts of the movement (start, peak, or end) could not be identified • No reaction was produced by the participant in response to tightrope walker's movement
Ego-centered response	The participant reacted to the TW's tilts by leaning to his own left when the TW leans to his right (or to his right when the TW leans to his left), indicating that he kept an ego-centered perspective and an embodied self-location
Hetero-centered response	The participant reacted to the TW's tilts by leaning to his own right when the TW leans to his right (or to his left when the TW leans to his left), indicating that he adopted an hetero-centered perspective and a disembodied self-location
Ambiguous movement	The participant reacted to the TW's tilts with an ego-centered or hetero-centered response before changing his response by leaning to the other side
**Movement's characteristics**	**Definition**
Amplitude of the participant's movement	The amplitude of the movement is the difference between the peek and the beginning of the movement
Length of the participant's movement	The length of the movement is the difference between the end and the beginning of movement
Latency of the annotated participant's movement	The latency of the movement is calculated on the difference between the TW movement start and the participant movement start
Overshoot	The overshoot corresponds to the delta between the amplitude of the movement and the amplitude of the TW's movement
Overlength	The overlength corresponds to the delta between the length of the movement, and the length of the TW's tilt or movement

### Statistical analysis

Statistical analyses were performed using R Software, Version 2.12.2. For every test, the level of significance, alpha, was fixed at 5%. To assess the number of tagged movements, we used a binomial linear model including age, sex, and group factors as explicative variables. For each trial, the tagged movement is a binary variable (“success” or “failure”). Thus, each experiment is a Bernouilli trial, and the number of successes (the sum of tagged movements of all trials, for each subject) can be modeled using a linear binomial model. A power calculation with *N* = 67, a significance level = 0.05, and an effect size = 0.15 (“medium” according to Cohen's, 1988 guidelines) yields a power of 73%.

Taking only into account the tagged movements, we assessed the following dependant variables using Generalized Linear Mixed Model (GLMM; lme4 package): latency, amplitude, length, overshoot, and overlength. These dependent variables have been modeled using longitudinal two level (patient and trial) mixed models. To the extent that we only used 5 models, we considered that no corrections of *p*-values were necessary.

The following explicative variables were entered in each model: developmental age, type of group (TD vs. DCD vs. ASD), the orientation of the TW (front vs. back-facing), gender, tasks (NI vs. OBT guided task), and trail number (1–7). For each dependant variable, the normal distribution was checked. In order to analyze the frequency of OBT, and taking only into account two types of responses (ego-centered and hetero-centered), the total amount of OBT was calculated for each subject. We used (1) a GLMM model including the following variables: developmental age, sex, and group (TD vs. DCD vs. ASD), orientation (front-facing and back-facing), and (2) a logistic regression for each orientation separately (face and back-facing) in order to investigate the association of the following predictors: age, sex, and group.

## Results

### Number of tagged movements and own-body-transformations (OBT)

All the three groups had a majority of trials with participant's interactive movements that were annotated. However, imitation was ambiguous or not performed in 19% (for TD), 25% (for DCD), and 39% (for ASD) of the TW movements. As a consequence, the percentage of tagged movements that was only performed on succeeded imitation increases between ASD group (61%) and the control group (81%); the DCD group having an intermediate position with 75% of tagged movements. The number of tagged movements (average, mean, and max) per group and per trial is given in Table 3. Binomial model showed several significant effects: (i) the number increased with age [*b* = 0.09, Standard error (SE) = 0.01, *p* < 0.001], (ii) it was higher in children with DCD and with TD compared to children with ASD (respectively, *b* = 0.58, *SE* = 0.1, *p* < 0.001; *b* = 1.03, SE = 0.09) *p* < 0.001); (iii) it was higher in TD group compared to children with DCD (*b* = 0.45, *SE* = 0.11, *p* < 0.001). In addition, we found that the proportions of tagged movements for the front-facing and back-facing orientations were homogeneous across the groups allowing further analyses.

**Table 3:** Number of tagged movements per trial for each group of participants.

	**TD (*****n*** = **36)**		**ASD (*****n*** = **25)**		**DCD (*****n*** = **14)**	
	**Average number of tagged movements**	**Min; Max number of tagged movements**	**Average number of tagged movements**	**Min; Max number of tagged movements**	**Average number of tagged movements**	**Min; Max number of tagged movements**
Trial 1	5,78	4; 6	4,08	0; 6	4,57	0; 6
Trial 2	5,75	4; 6	4,24	0; 6	5,21	0; 6
Trial 3	5,61	4; 6	4,60	0; 6	5,21	0; 6
Trial 4	5,64	4; 6	4,36	1; 6	5,36	0; 6
Trial 5	5,61	3; 6	4,28	0; 6	5,29	3; 6
Trial 6	5,61	3; 6	4,32	0; 6	5,36	2; 6
Trial 7	5,64	2; 6	4,04	0; 6	5,57	4; 6
Total	39,64		29,92		36,57	

**Figure 2:**
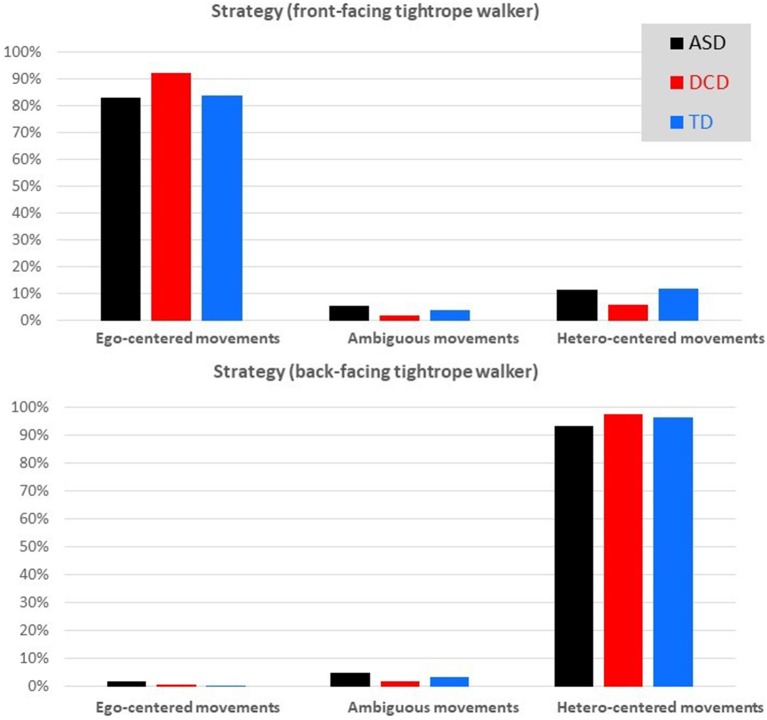
The type of annotated movements (mirror symmetry vs. mixed vs. OBT) according to groups (ASD vs. DCD vs. TD) and orientation of the tightrope walker (front-facing vs. back-facing). ASD, autism spectrum disorder; DCD, developmental coordination disorder; TD, typical development.

Considering the number of tagged movements only, the type of strategies for each of the three groups considering separately the orientation (front-facing vs. back-facing) of the TW is shown in Figure 2. In front-facing, the majority of tagged movements were ego-centered, and conversely, in back-facing, the majority of tagged movements were hetero-centered. When the TW is back-facing, the task was very well performed for all groups, with a percentage of ET of almost 100% for TD and DCD children and 97% for ASD children.

**Figure 3:**
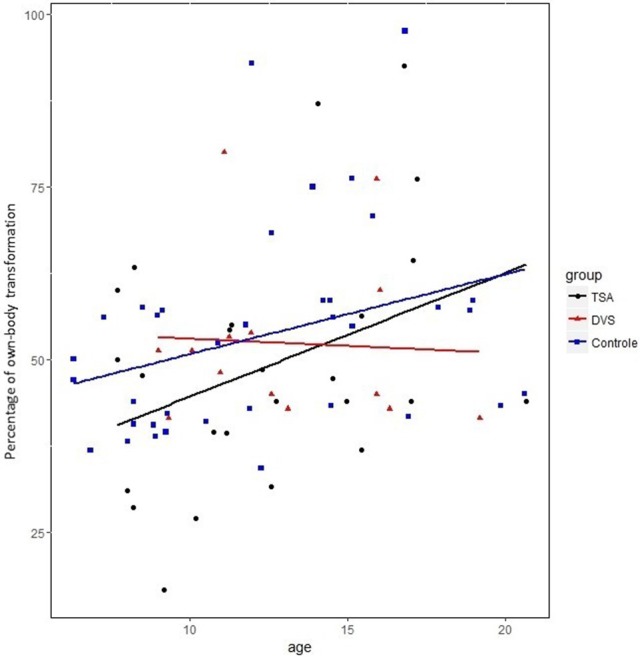
Own-body-transformation scores according to age and groups. ASD, autism spectrum disorder; DCD, developmental coordination disorder; TD, typical development.

**Figure 4:**
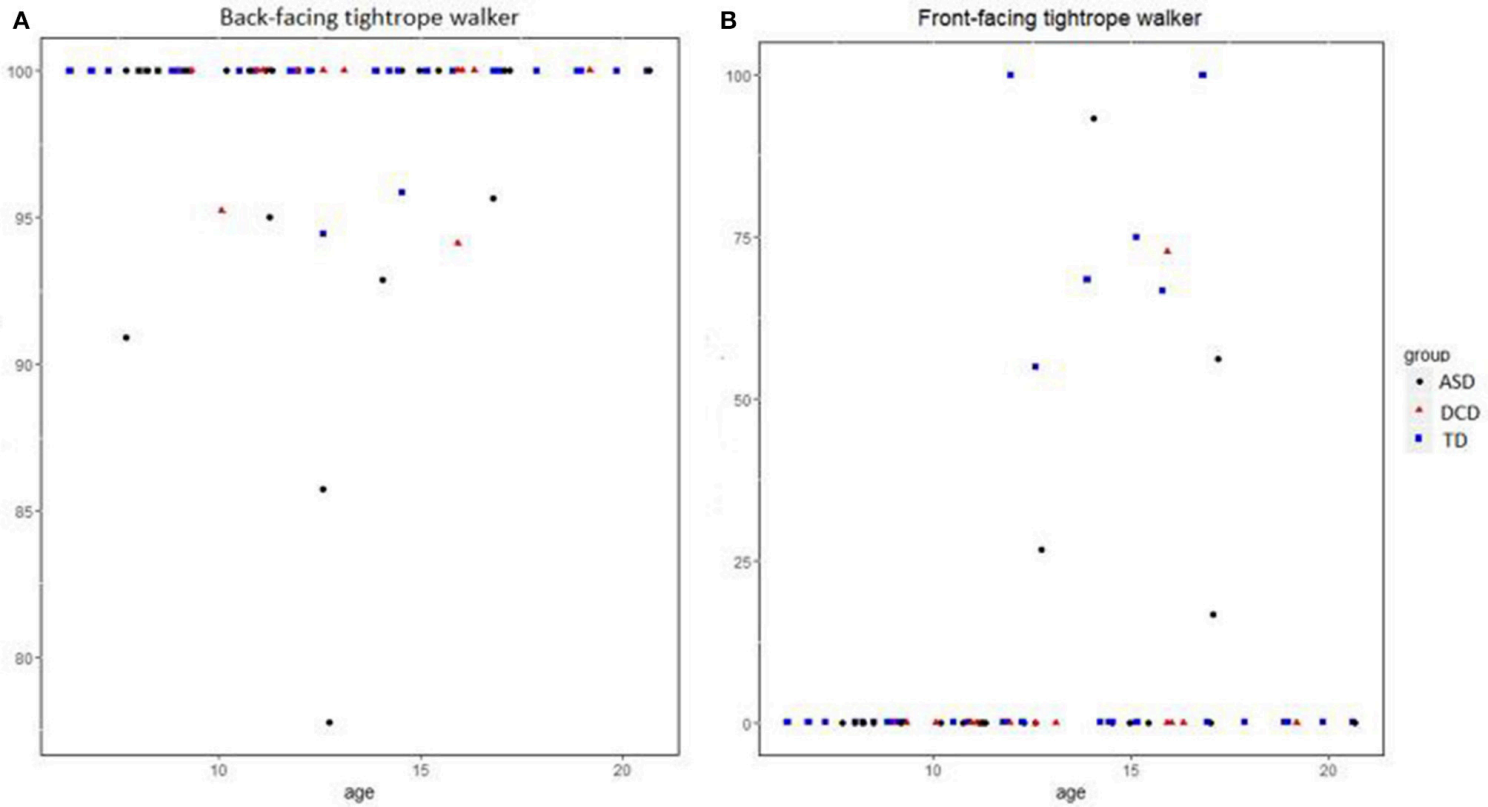
Own-body-transformation scores according to age and groups when the tightrope walker is in the back-facing orientation **(A)**. Own-body-transformation scores according to age and groups when the tightrope walker is in the front-facing orientation **(B)**. ASD, autism spectrum disorder; DCD, developmental coordination disorder; TD, typical development.

Taking into account both front and back-facing orientations, binomial GLMM confirmed that the rate of OBT increased with age (*b* = 0.23, *SE* = 0.10, *p* = 0.03) and that there was a major effect of the TW's orientation (*b* = −11.7, SE = 0.76, *p* < 0.001; Figure 3). The rate of OBT was significantly higher when the tightrope was back-facing (Cf. Figure 4A) than when it is front-facing (Cf. Figure 4B).

According to this last result, we performed a logistic regression by groups and for each orientation. When the tightrope is front-facing, the logistic regression showed that: (i) perspective taking was significantly and positively associated with age (*b* = 0.19, *SE* = 0.025, *p* < 0.001), (ii) boys had a higher rate of OBT than girls (*b* = 0.69, *SE* = 0.21, *p* < 0.001), (iii) DCD had a lower rate of OBT than ASD (*b* = −0.77, *SE* = 0.31, *p* = 0.013), and (iv) a lower rate of OBT than TD (*b* = −1.04, *SE* = 0.3, *p* < 0.001). The ASD group did not perform significantly worse than the TD group (*b* = −0.27, *SE* = 0.21, *p* = 0.19). When the tightrope is back-facing, we did not find an age or a gender effects (*b* = 0.03, *SE* = 0.08, *p* = 0.73; *b* = 0.11, *SE* = 0.7, *p* = 0.87, respectively). We found, however, that TD children had a higher rate of OBT than children with ASD (*b* = 1.95, *SE* = 0.82, *p* = 0.017), and that there was no difference between TD and DCD children (*b* = −1.05, *SE* = 1, *p* = 0.3).

### Characteristics of the participant tagged movements

Table 4 shows the characteristics of the tagged movements in each of the three groups. Assessing the variables associated with the characteristics of the movement the GLMM model showed the following:

**Table 4:** Description of the characteristics of tagged movements in the three groups.

**Movements**	**ASD (*****N*** = **25)**		**DCD (*****N*** = **14)**		**TD (*****N*** = **36)**	
**Mean (**±***SD*****)**						
Amplitude (degree)	*n* = 748	46.26 (±19.1)	*n* = 512	33.66 (±16)	*n* = 1,427	33.6 (±13.6)
Latency (second) Ego-centered Hetero-centered Ambiguous	*n* = 748 *n* = 323 *n* = 385 *n* = 40	0.67 (±0.91) 0.71 (±1.22) 0.63 (±0.35)	*n* = 512 *n* = 242 *n* = 260 *n* = 10	0.64 (±0.29) 0.69 (±0.28) 0.59 (±0.28)	*n* = 1,427 *n* = 604 *n* = 769 *n* = 54	0.55 (±0.25) 0.55 (±0.24) 0.53 (±0.22)
Length (second)	*n* = 746	1.87 (±0.6)	*n* = 512	1.85 (±0.44)	*n* = 1,427	1.76 (±0.55)
Overlength (second)	*n* = 746	0.36 (±0.61)	*n* = 512	0.34 (±0.37)	*n* = 1,427	0.3 (±0.35)
Overshoot (degree)	*n* = 748	50.1 (±43.47)	*n* = 512	49.59 (±35.3)	*n* = 1,427	46.11 (±35.3)

*N, number of participants; n, number of trials*.

#### Latency

Latency was significantly lower with age (*b* = −0.25, *SE* = 0.004, *p* < 0.001) in TD group compared to ASD (b = −0.14, *SE* = 0.04, *p* < 0.001) and when the tightrope is front-facing vs. back-facing (*b* = −0.065, *SE* = 0.02; *p* < 0.001). Since in previous adult studies, latency significantly increased in hetero-centered perspective tacking as it may require a spatial rotation (Thirioux et al., 2014a). We also tested latency according to hetero-centered perspective and found that latency significantly increased when participants used a hetero-centered perspective.

#### Amplitude

Amplitude was lower in TD and DCD groups compared to ASD group (*b* = −13.32, *SE* = 2.67, *p* < 0.001; *b* = −13.85, *SE* = 3.41, *p* < 0.001; respectively); no significant difference was found between DCD and TD group.

#### Length

Length was higher in front-facing vs. back-facing TW orientations (*b* = 0.016, *SE* = 0.007, *p* = *p* = 0.046) and in the OBT-guided task vs. the no instruction task (*b* = 0.03, *SE* = 0.013, *p* = 0.032).

#### Overlength

The overlength was significantly and negatively associated with the trials number (*b* = −0.012, *SE* = 0.006, *p* = 0.048).

#### Overshoot

A histogram of the distribution of overshoot showed that this variable was a mixture of two distributions. A graphical exploratory analysis revealed that it was due to the dependent variable *orientation* (i.e., front *vs*. back-facing). Indeed, in the case of the tightrope back-facing, the values of overshoot were very small in comparison with front-facing values. Thus, an analysis was conducted for each orientation separately (front-facing and back-facing). None of the predictors were found to be significant, so it seems that only the TW's orientation was associated with the overshoot.

## Discussion

In this study, we explored the development of behavioral OBT and its potential alteration in ASD and DCD children. In the context of our dynamic and interactive task, to disentangle the contribution of visual- spatial impairments associated with motor coordination impairments, we compared a group of children with ASD, a group of children with DCD, and a group of TD children matched for developmental age. We also analyzed through semi-automatic quantification of movements how motor performance evolved during our experimental task. Following Thirioux et al.'s study (Thirioux et al., 2014a,b), this is the first clinical study on children assessing OBT from a semi-ecological point of view through an imitation task.

### Typical developing children

We first found *a strong developmental age* effect as evidenced by the increase of tagged movements with age, by the decrease of the child response (latency), and by the improvement of OBT (Figure 3). This improvement of OBT with age is more significant with the front-facing orientation of the TW which involves for the participant a mental rotation in order to adopt a hetero-centered perspective. This age effect concerning the ability to move out from an egocentric point of view is consistent with Piaget and Inhelder's theory (Piaget and Inhelder, 1997), even though for Piaget, this ability begins earlier, a conclusion supported by Frick et al. (2014). Studying the developmental time course of ego- and allo-centric perspectives, Bullens et al. (2010) tested children using a virtual reality adaptation of the StarMaze task (Iglói et al., 2009): from a predominant egocentric strategy, children progressively develop the spontaneous use of an allocentric strategy from age 10. Belmonti et al. (2014), using the Magic Carpet which is a spatial memory task in a navigational space, confirmed this cognitive shift occurs between 10 and 11 years.

As Belmonti showed among adults, we found that boys had higher OBT scores than girls. This finding is consistent with Baron-Cohen (2010) who distinguishes the better ability of the male brain to “systematize” which includes tests of mental rotation or card reading, from the ability of the female brain which he describes as superior in terms of empathizing capacity (sharing and sensitivity). The better scores obtained by boys compared to girls in OBT are also in line with Lange-Küttner and Bosco (2016) who found better spatial representation abilities of boys compared to girls. The authors explained this superiority by boys having a greater experience in ball games, i.e., their experience of being and acting within in a spatial field rather than girls.

### Patients with neuro-developmental disorder

In the back-facing orientation of the TW, OBT does not require self-mental rotation and is completely successful irrespective of age in TD and DCD groups. In this orientation which does not require visual- spatial abilities of the participants, the rate of OBT in ASD group is very high but significantly lower than that of TD group. Moreover, the number of tagged movements in ASD group was significantly lower compared to the TD and DCD groups. These results combined with those obtained through systematic measures of the movement's quality in ASD (higher latency and higher amplitude) are in line with Wild et al. (2012) showing that autistic adolescents were less sensitive to the duration, velocity, and vertical amplitude of observed actions during this goalless imitation task. This is confirmed by Edwards who argued that impairments in imitation of children with ASD include differences between mimicry behavior (copying the form, i.e., detailed kinematic features of an observed action) and emulation tasks (i.e., involving the copy of the explicit goal of an observed action) for which autistic participants tend to be proficient (Edwards, 2014). This suggests that a proportion of motor responses of participants of ASD group was inadequate in the current imitation paradigm. This could also be explained by the impairments in visual-spatial processing regarding dynamic information analysis described in this population (Bertone et al., 2005). These impairments are in line with deficits in motion perception tasks (Annaz et al., 2012) including biological motion cues such as body gestures and actions (Kaiser and Pelphrey, 2012). All the more so, children with ASD performed well on OBT: in front-facing orientation, the ASD group performed significantly better than the DCD group and no differences were found between the ASD and TD group's performances. These findings seem to be incongruent with the impairments in OBT described in individuals with ASD (Pearson et al., 2014) unless one considers the differences between the two tasks. Pearson et al. (2014) used an experiment where the participants had to make a decision about images and respond orally whereas our paradigm involved participants who were moving and imitating a partner allowing a spatial disembodied self-location through these interactions.

In addition, the ability to perform a mental transformation requires the suppression of conflicting frames of reference by inhibiting one's own dominant perspective (first-person perspective) between two spatial frames of reference. This begs the question of whether OBT ability is associated with executive functioning, more specifically with inhibitory control. Frick and Baumeler (2016) found a significant correlation between PT and inhibitory control, both abilities showing developmental progression into childhood (Davidson et al., 2006; Aïte et al., 2016). ASD's performances in inhibitory control yielded contrary results in the literature (for a review, see Xiao et al., 2012): several studies reported significant impairments of inhibitory control in individuals with autism, whereas other studies suggest that there is no difference between subjects with autism and healthy controls with regard to inhibition measures.

DCD group's performances were equivalent to those of TD group concerning the number of tagged movements, the rate of OBT in back-facing orientation, and the amplitude of their movements. However, their performance in OBT was lower in front-facing orientation than that of TD and ASD participants. Since DCD had better movement quality than ASD (given the similar tagged movements and amplitude with TD), we assume that visual-spatial abilities are key here, since this group was specifically handicapped by visual-spatial impairments. The task we used was extremely demanding in terms of spatial mental rotation and orientation of participant's own body in space during the front-facing orientation.

Finally, we found few significant effects regarding the instruction given during the tasks (no instruction task vs. OBT-guided task), where the participants were instructed explicitly to imagine their body in the position of the TW's body and then driven to adopt a hetero-centered perspective. In adults, the OBT-guided task may facilitate OBT. The fact that we also found an increase of the amplitude and of the length of the movements during the OBT-guided task may appear contradictory unless one considers that the facilitation in OBT in the experiment implicates employing more inhibitory resources. This idea of cognitive cost introduces a cognitive load, which we did not directly manipulate in our experiment.

### Study limitations

The results of the current study should be interpreted according to the study limitations. First, the number of participants in the three groups was somewhat restricted and particularly in DCD group compared to the other two groups. Second, visual-spatial abilities and cognitive control functions of participants were not evaluated, knowing that both are tested in such a task. Third, the fact that there are mainly males among participants with ASD is both consistent with literature highlighting a higher rate of ASD diagnosis in males than females and the source of a gender bias. However, variability among individuals with respect to gender, especially in a developmental perspective, was rarely taken in consideration. Finally, our results are carried on the basis of a manual annotation of children's movement trajectories whose necessarily subjective character must be taken into account.

## Conclusion

We found a developmental age effect for the mental body transformation abilities during this dynamic imitative task that appeared relatively difficult for children with neurodevelopmental disorders as well as for TD children. Indeed, we found a majority of ego-centered movements when the task required the participant to perform a mental rotation in order to adopt a hetero-centered perspective. ASD group performances were better than those of DCD group, and to some extent close to those of TD group. However, a proportion of ASD participant's motor responses were more ambiguous and hardly readable, which seems to reveal specific impairments concerning kinematic features of an observed action. Using automatic quantifications of movement, further studies should investigate motor imitation quality in ASD from a kinematic point of view.

## Author contributions

JX is one of the promoters of the research project and has participated in its drafting and proofreading. SG led the experimental part of our research with children and adolezcents; she also participated to the writing of our draft. SA, MZ, and MC have built the experimental setup of our research. They participated also to the drafting of our article. AB, DC, and FV are the promoters of the research project in which this article belongs. AB is the author of a part of the theoretical foundations on which relies our research. They all three have also participated to the proof reading of our draft.

### Conflict of interest statement

The authors declare that the research was conducted in the absence of any commercial or financial relationships that could be construed as a potential conflict of interest.
