# Supplementary material for: Behavioral Own-Body-Transformations in Children and Adolescents With Typical Development, Autism Spectrum Disorder, and Developmental Coordination Disorder
Source: Front Psychol. 2018 May 25;9:676. doi: 10.3389/fpsyg.2018.00676 (PMC5981221; doi:10.3389/fpsyg.2018.00676)
Supplement: Supplementary file 1 [file Presentation_1.pdf]

Supplementary materials

1. Table S1: Definitions of the events in the trajectory curve

Event name	Event definition
Movement	A set of frames during which the bar inclination angle significantly varies. It comprises at least one up or down trajectory and one return towards the initial position (See Figure S1).
Tagged movement	A participant's movement in response to a tightrope walker's movement. Therefore, a participant's movement initiated before, or initiated after a first response but still before the next tightrope's walker movement the tightrope walker's movement, cannot be a tagged movement (See Figure S3).
Movement start	The movement start is the accurate frame during which a movement is clearly initiated. If the bar was maintained still before the response, the response starts at the frame during which this stability is altered. If regular swings preceded the response, the response start is the frame marking the beginning of the angle variation becoming significantly more important in comparison with the previous swings. In this case, the movement must last longer than the previous swings in order to be considered as a response.
Movement peak	The movement peak is the accurate frame of the response that has the higher bar angle value. If several frames in one response follow this definition, the response peak is the frame first occurring chronologically.
Movement end	The movement end is the accurate frame during which a response is clearly finished. If the bar was maintained still before the response, the response ends at the frame during which this stability is recovered. If regular swings preceded the response, the response start is the frame marking the beginning of the angle variation coming back to those regular swings.

2. Examples of recorded participants movements and tightrope walker movements

Figure S1. Both the participant and the tightrope walker remain still (yellow box).

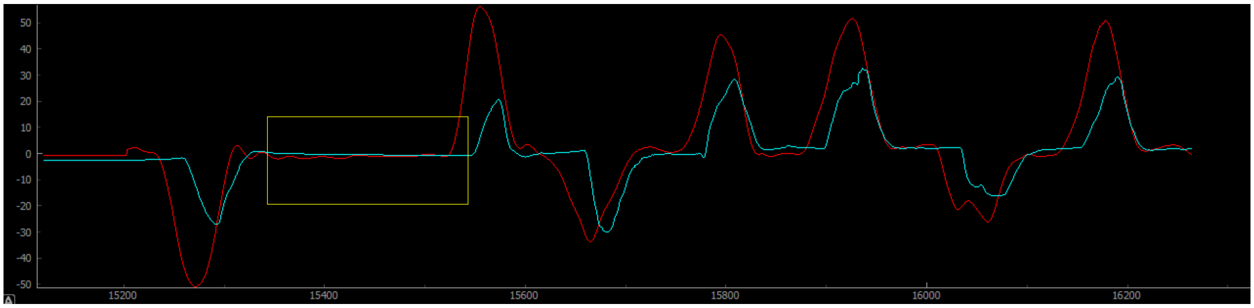

Figure S2. The participant moves with a regular swing (top) or does not come back to his initial position after a movement (down) (yellow box).

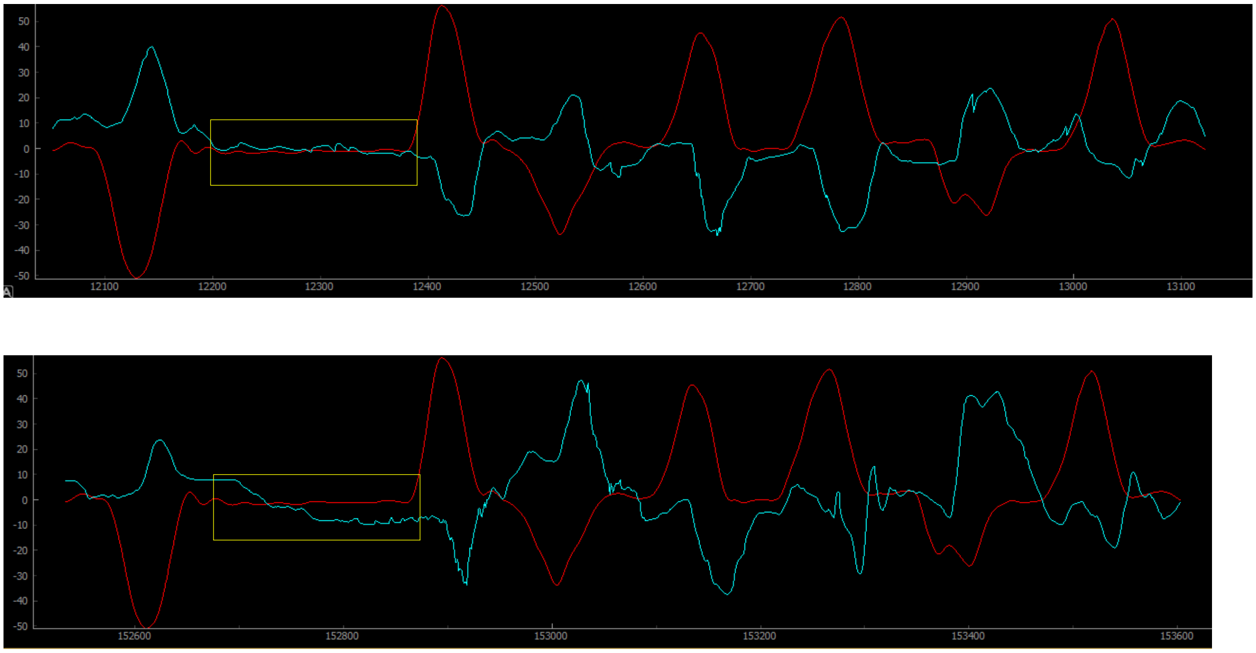

Figure S3. The participant makes a movement which is not a response to the tightrope walker movement (yellow box).

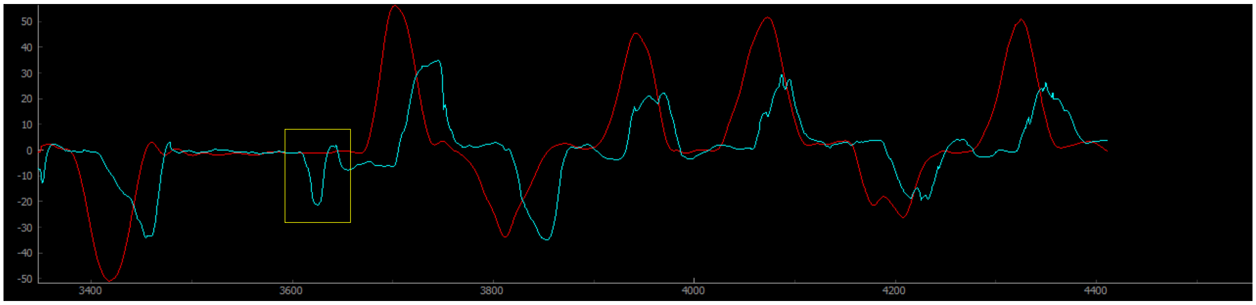

3. How annotations were conducted

Figure S4. Annotations of a participant response starts during a trial (yellow lines).

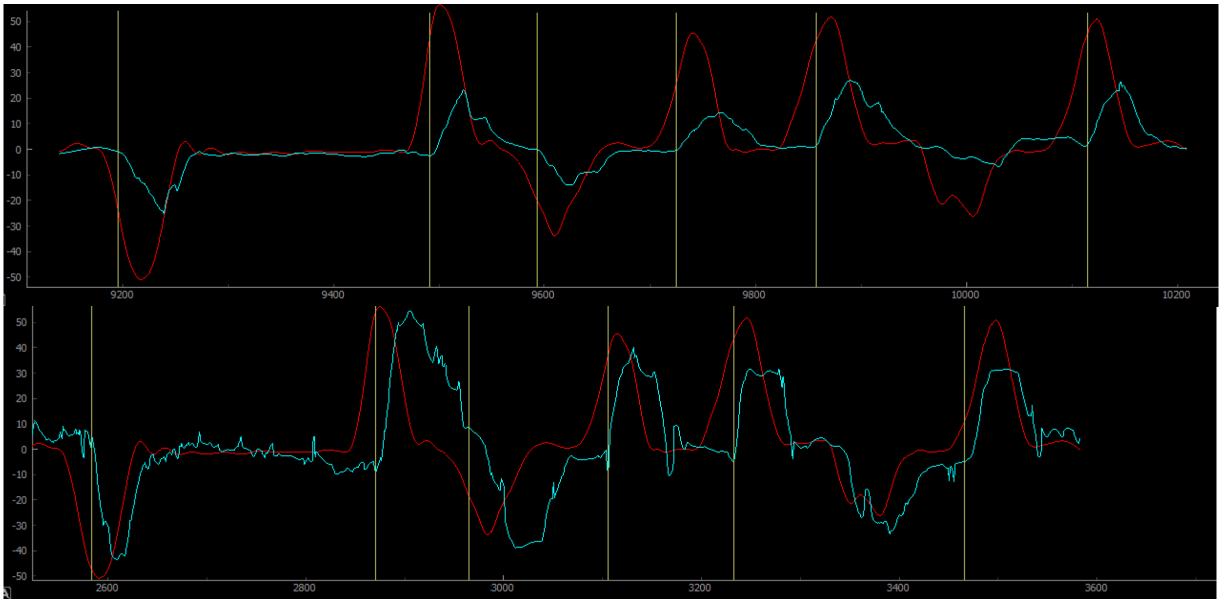

Figure S5. Annotations of a participant response peaks during a trial (yellow lines).

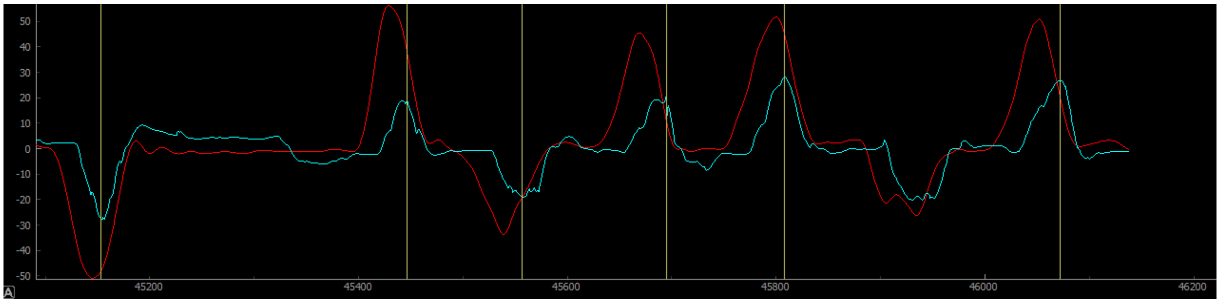

Figure S6. Annotations of a participant response ends during a trial (yellow lines).

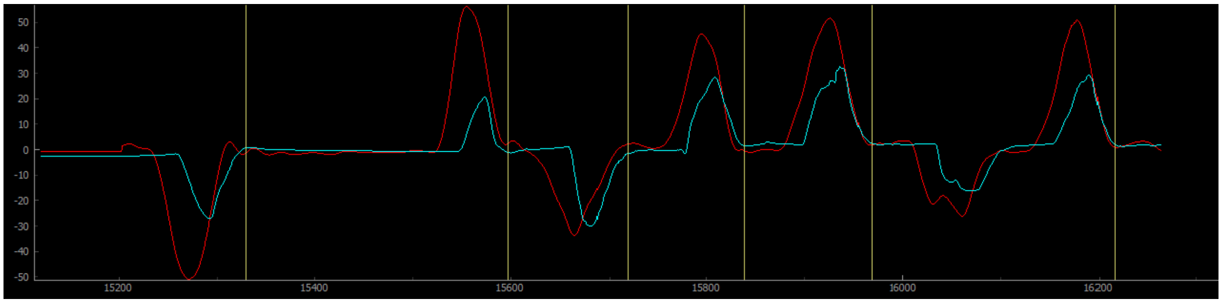

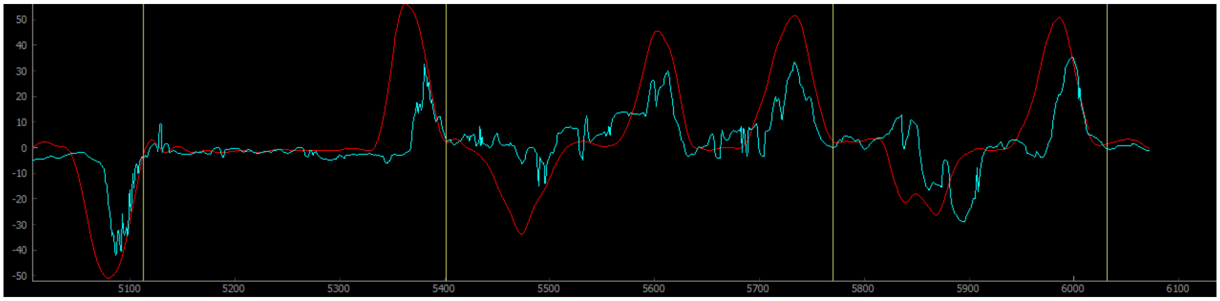

Figure S7. Examples of participant responses that were considered as response that could not be annotated (yellow box) whether there was a complete lack of response (top), or whether it was impossible to determine every element of the response (down, in this case the participant response end could not be clearly determinated).

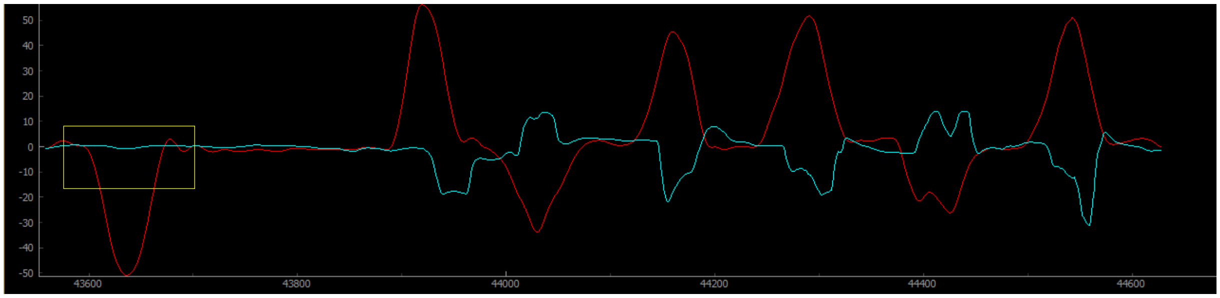

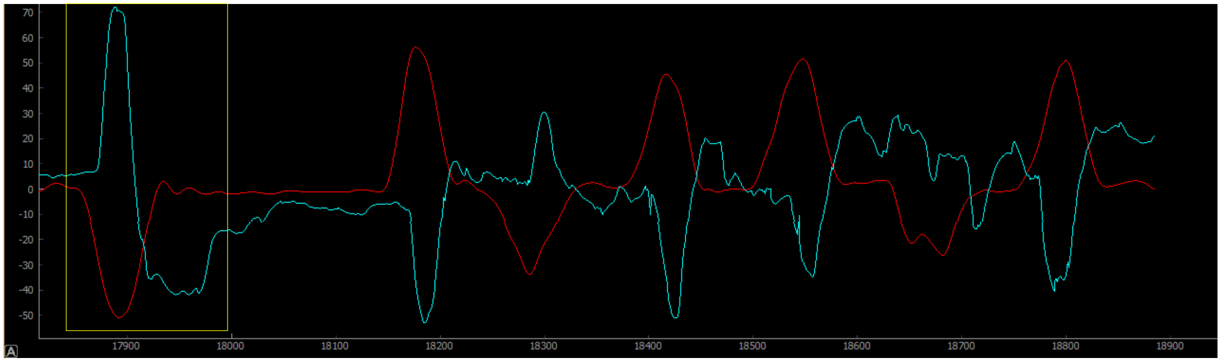

Figure S8. Annotations of participant perspective taking response (yellow box) according to the tightrope walker movements: ego-centered response (top), hetero-centered or mirrored response (middle), ambiguous response (down).

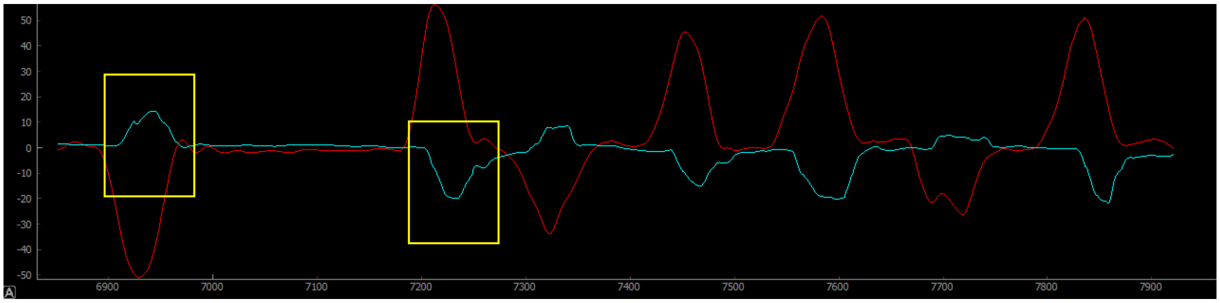

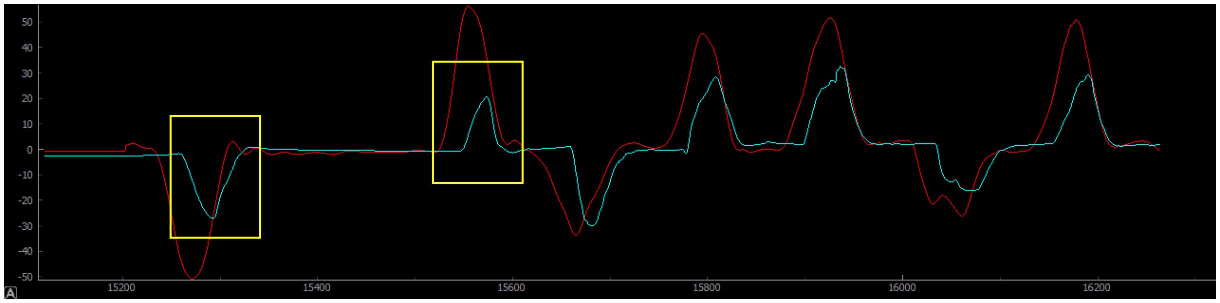

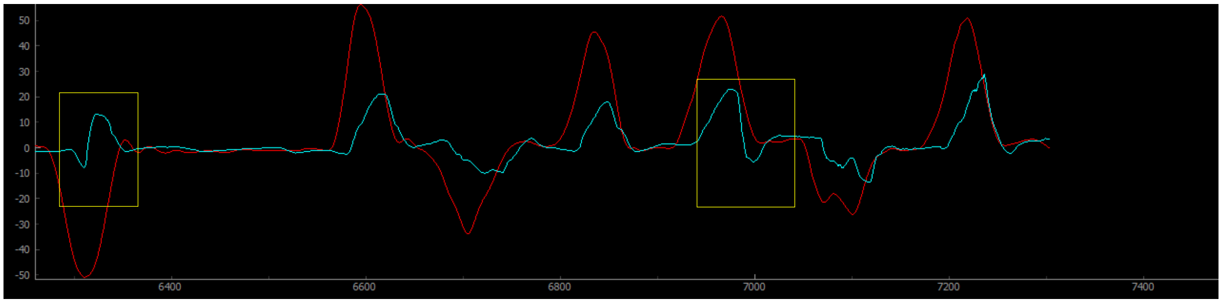

4. Summary of the precautions taken in order to obtain accurate Kinect records

- The experimenter was situated in a part of the room the Kinect could not perceive, so he wouldn't be an eventual disturbance.
- Still, the experimenter could see the participant and the figure of the participant as recorded by the Kinect during the whole time of the test. The experimenter had to make sure that the figure was congruent with the participant before starting the test and during the test. If the figure was not correctly recorded, the test would not begin until this problem was resolved. The participant could not see his figure at any time of the test.
- Since the participants' clothing had an effect on the quality of the recording, we asked them to wear plain colored clothes. We also asked them to roll-up their sleeves before starting the test.
- Participants' hands were placed on some precise marked spots on the bar, separated by a 78-centimeter space, and the bar was painted black, so that the Kinect could more easily differentiate the bar from the participants' hands.
